# Reframing emergency preparedness from facility survival to laboratory animal welfare

**DOI:** 10.1186/s42826-026-00295-5

**Published:** 2026-09-08

**Authors:** Seung Yeon Kim, Ji Min Lee, Seung Hyeok Seok

**Affiliations:** 1 Animal Research Facility, Science, Institutional Vaccine Institute, Seoul, Republic of Korea; 2 Institute of Environment and Safety, Seoul National University, Seoul, Republic of Korea; 3 Macrophage Lab, Department of Microbiology and Immunology, and Institute of Endemic Disease, Seoul National University College of Medicine, Seoul, Republic of Korea; 4 Department of Biomedical Sciences, Seoul National University College of Medicine, Seoul, Republic of Korea; 5 Cancer Research Institute, Seoul National University College of Medicine, Seoul, Republic of Korea

**Keywords:** Emergency preparedness, Animal welfare, Disaster management, 3Rs, Laboratory animal facilities

## Abstract

**Background:**

Natural disasters, infrastructure failures, and infectious disease outbreaks can disrupt the operations of laboratory animal facilities and threaten both research continuity and animal welfare. Previous events, including Tropical Storm Allison and the Great East Japan Earthquake, demonstrated the vulnerability of laboratory animal populations during emergencies and highlighted the need to incorporate animal welfare considerations into emergency preparedness planning. The aim of this study was to identify key elements of emergency preparedness programs for laboratory animal facilities and to propose a framework to support the development of practical Standard Operating Procedures (SOPs) in which animal welfare considerations are integrated into facility operations and decision-making processes during emergencies.

**Results:**

Regulatory frameworks and guidance documents from the United States, Europe, Canada, and Korea were reviewed and compared to identify approaches applicable to laboratory animal facility management. The analysis included definitions of emergencies, core program components, veterinary oversight systems, and decision-making structures across these four regulatory contexts. Despite differences in regulatory approaches and institutional contexts, common elements were identified and organized into three functional domains: continuity of facility operations, protection of animal welfare, and institutional response capacity. Based on these findings, we propose an integrated emergency preparedness framework and a stepwise operational checklist intended to facilitate the integration of welfare priorities into emergency planning and response.

**Conclusions:**

Emergency preparedness should not be regarded solely as an operational requirement. In laboratory animal facilities, it should be understood as an extension of the animal welfare program that, while prioritizing personnel safety and institutional resilience, also seeks to minimize animal suffering and preserve the value of ongoing research when normal operating conditions cannot be maintained.

**Supplementary Information:**

The online version contains supplementary material available at https://doi.org/10.1186/s42826-026-00295-5.

## Background

Laboratory animal facilities have increasingly faced emergencies that threaten the continuity of animal care and research operations, including natural disasters, infrastructure failures, prolonged interruptions of power or water supply, and staffing shortages associated with infectious disease outbreaks [1–6]. These events present substantial challenges for facilities that rely on tightly controlled environmental systems and continuous personnel support to maintain animal health and welfare [7–9].

Animals housed in laboratory animal facilities are maintained within cages and barrier systems and depend heavily on facility infrastructure and human intervention for their survival and welfare [6, 10]. Consequently, failures of essential systems such as power, Heating, Ventilation, and Air Conditioning (HVAC), or water supply can directly compromise animal health and welfare. Animal welfare concerns arising during emergencies should therefore be regarded not as exceptional events, but as foreseeable risks that warrant advance planning and institutional preparedness [7, 11, 12].

Past emergencies illustrate the vulnerability of laboratory animal populations during major disruptions. During Tropical Storm Allison in 2001, flooding resulted in the loss of approximately 35,000 laboratory animals despite the existence of emergency plans [9]. Similarly, following the Great East Japan Earthquake in 2011, severe infrastructure damage at a major breeding facility led to the humane euthanasia of approximately 14,000 animals [13]. These events demonstrate that substantial animal welfare consequences can arise when critical facility functions cannot be maintained [6, 10].

The importance of emergency preparedness is increasingly reflected in international regulatory frameworks. In the United States, the Animal Welfare Regulations (AWR) require contingency planning for covered species, while the Public Health Service (PHS) Policy and the Guide for the Care and Use of Laboratory Animals emphasize disaster planning and emergency preparedness [7, 11]. In European Union (EU), Directive 2010/63/EU, as amended by Commission Delegated Directive (EU) 2024/1262, explicitly requires effective contingency plans to safeguard animal health and welfare in the event of failure of essential husbandry systems [14]. The Canadian Council on Animal Care (CCAC) similarly provides guidance for institutional crisis management programs. However, the regulatory approaches, implementation mechanisms, and degree of enforceability differ across jurisdictions [15].

Although emergency preparedness is increasingly recognized within regulatory and professional frameworks, practical approaches for integrating animal welfare considerations into institutional preparedness and emergency decision-making remain insufficiently examined. Furthermore, while the principles of Replacement, Reduction, and Refinement (3Rs) are widely accepted as the foundation of ethical animal research, their application under emergency conditions, where resources may be limited and rapid decisions are required, remains poorly defined [16]. Decisions regarding continued animal care, relocation, research continuation, or humane euthanasia may therefore need to be made under considerable uncertainty. In the absence of predefined principles and procedures, consistent and accountable decision-making can be difficult to achieve [17].

This study compares emergency preparedness regulations and guidance documents from the United States, Europe, Canada, and Korea to identify common elements of emergency preparedness programs for laboratory animal facilities. Based on this analysis, an Integrated Emergency Preparedness Framework and a practical checklist were developed to support institutional preparedness planning and decision-making during emergencies.

## Methods

### Study design and scope

This study used a comparative analysis of regulatory and guidance documents related to emergency preparedness in laboratory animal facilities in the United States, Europe, Canada, and Korea. The analysis focused on how emergencies are defined and how core elements of institutional emergency preparedness programs are addressed across jurisdictions. For the purposes of this study, an emergency was defined as any event that significantly disrupts normal facility operations and adversely affects animal care, research activities, or institutional response capacity. Incidents involving pathogen release or containment breaches were excluded because they require separate biosafety and biosecurity response frameworks.

### Regulatory and guidance document review

Relevant literature, regulations, and guidance documents addressing emergency preparedness in laboratory animal facilities were identified for the United States, Europe, Canada, and Korea. The reviewed sources included the Animal Welfare Regulations, Public Health Service Policy, the Guide for the Care and Use of Laboratory Animals, Directive 2010/63/EU, FELASA recommendations, CCAC guidelines, and relevant Korean regulations and guidance documents, as well as published literature addressing emergency preparedness and disaster management in laboratory animal facilities [7, 11, 14, 15]. Additional relevant literature and guidance materials were identified through references and related resources associated with the reviewed regulatory and guidance documents. The identified sources were comparatively analyzed across jurisdictions to examine how emergency situations were defined and to identify the core operational and organizational elements addressed in emergency preparedness programs.

The analysis focused on recurring operational and organizational elements of emergency preparedness, including contingency planning, veterinary involvement, emergency communication, personnel training, resource management, and procedures for maintaining animal care during service disruptions.

### Development of the integrated emergency preparedness framework and checklist

Based on the recurring elements identified through comparative analysis, elements addressing similar operational or organizational functions of emergency preparedness programs were organized into functional domains: facility operational continuity, animal care and welfare, and institutional response capacity. These domains formed the basis of the Integrated Emergency Preparedness Framework. To integrate these elements into a practical structure for laboratory animal facilities, a four-step framework for emergency preparedness and welfare-informed decision-making was developed. The framework consisted of emergency definition, risk identification and assessment, preparedness planning, and operational decision-making for animal management during emergencies.

The same comparative analysis was used to develop a practical emergency preparedness checklist for laboratory animal facilities. The checklist was organized into six domains: governance and responsibility structure, animal care and veterinary oversight, infrastructure and resource management, training and program validation, communication and coordination, and security and personnel safety. Checklist items were categorized as either “Must” or “Should” based on the consistency with which they were identified across the regulatory and guidance frameworks reviewed. “Must” items represented foundational elements consistently identified across multiple frameworks, whereas “Should” items represented additional measures supported by selected guidance documents or current best practices. This classification does not imply legal enforceability but rather reflects the relative consistency, practical importance, and strength of support identified across the reviewed frameworks.

## Results

### Regulatory frameworks for emergency preparedness in laboratory animal facilities

#### Definitions of emergency situations in regulatory frameworks

A comparative analysis of emergency preparedness regulations and guidance documents demonstrated that emergencies are generally recognized as events capable of disrupting essential functions required for animal care and facility operations, thereby necessitating advance planning at the institutional level (Table 1). Regulations and guidance documents from the United States, Europe, Canada, and Korea each recognize preparedness against a range of emergency scenarios as an institutional responsibility. The regulatory mechanisms differ across jurisdictions, and the classifications presented in Table 1 reflect the degree to which emergency preparedness requirements are explicitly articulated within the reviewed documents.

**Table 1:** Comparison of regulatory frameworks for emergency preparedness in laboratory animal facilities

Organization	Regulatory instrument	Title (Authority)	Regulatory level		Key Provisions
			Program requirement	Scope & Elements	
USA	Law	The Animal Welfare Regulations (USDA)	Mandatory (Must)	Mandatory (Must)	9 CFR 2.38(I) Contingency planning (l) Contingency planning. (1) Research facilities must develop, document, and follow an appropriate plan to provide for the humane handling, treatment, transportation, housing, and care of their animals in the event of an emergency or disaster (one which could reasonably be anticipated and expected to be detrimental to the good health and well-being of the animals in their possession).
	Guideline	Guide for the Care and Use of Laboratory Animals (PHS)	Mandatory (Must)	Recommendation (Should)	P35. Chapter 2. ANIMAL CARE AND USE PROGRAM - DISASTER PLANNING AND EMERGENCY PREPAREDNESS Facilities must therefore have a disaster plan.
EU	Law	Directive 2010/63/EU as amended by Commission Delegated Directive (EU) 2024/1262	Mandatory (Must)	Mandatory (Must)	ANNEX(1)(a)(iv)(d) Effective contingency plans shall be in place to ensure the health and welfare of the animals in the case of failure of essential husbandry elements.
	Guidance	FELASA recommendations for the veterinary care of laboratory animals	Recommendation (Should)	Recommendation (Should)	Every unit should also have an emergency plan for dealing with disasters or other emergencies such as attacks on property or personnel.
CCAC	Guideline	CCAC Crisis Management Program	Recommendation (Should)	Recommendation (Should)	The CCAC states that all institutions which use experimental animals have in place a crisis management program for their animal facilities and for their animal care and use program. This program should be developed in conjunction with any general institutional crisis management plan(s).
Republic of Korea	Guideline	Standard Operating Guidelines for the Institutional Animal Care and Use Committee (IACUC) (APQA)	Recommendation (Should)	Recommendation (Should)	The Committee shall possess general knowledge regarding the care of laboratory animals and the management of animal facilities, and shall conduct on-site inspections based on the fundamental principles set forth below, while reflecting the specific characteristics of the institution subject to inspection and relevant scientific evidence.The facility inspection checklist shall be prepared with reference to the Animal Experiment Facility Inspection Form provided in Chapter 6 or formats suggested by the most recent guidelines, and may be modified as appropriate to reflect the current status and characteristics of the institution. - Status of backup facilities for 24-hour animal testing facilities, measures to prepare for unexpected events such as fires and power outages, firefighting equipment and emergency power systems, and security alarms.
	Law	Laboratory animal act (KFDA)	Mandatory (Must)	Mandatory (Must)	Article 2 (Definitions) 3. The term “disaster” means infection in humans and animals, occurrence of an infectious disease, exposure to harmful substances and environmental pollution or such, due to animal testing Article 18 (Prevention of disasters) (1) Where an operator or an administrator of animal testing facilities conducts animal testing using substances or pathogens or such, which may cause any disasters, he or she shall take the necessary measures so as not to do any harm to humans and animals.

Despite this shared recognition, the regulatory approaches supporting emergency preparedness differed substantially among jurisdictions. In the United States, the Animal Welfare Regulations (AWR) explicitly require research facilities to develop and implement contingency plans for emergency situations, establishing preparedness as a legal obligation. The Public Health Service (PHS) Policy further reinforces this expectation through adherence to the Guide for the Care and Use of Laboratory Animals, which states that institutions must maintain disaster plans appropriate to their programs and emphasizes continuity of animal care during disruptions. In the European Union, emergency preparedness has recently been incorporated into the regulatory framework through Commission Delegated Directive (EU) 2024/1262, which amended Directive 2010/63/EU to require that effective contingency plans be established to safeguard animal health and welfare in the event of failures affecting essential husbandry elements. These emergency planning requirements were explicitly introduced into Annex III of Directive 2010/63/EU and will become applicable from December 2026. Complementing this legal framework, Federation of European Laboratory Animal Science Associations (FELASA) provides practical recommendations regarding veterinary involvement, emergency planning, and institutional preparedness [18]. Similarly, the Canadian Council on Animal Care (CCAC) recommends that institutions incorporate crisis management procedures into their animal care and use programs. In Korea, emergency-related considerations are addressed through institutional guidance documents and selected regulatory provisions; however, explicit contingency planning requirements focused on the continuity of routine animal care have not been established as a distinct and comprehensive regulatory framework.

Meanwhile, emergency-related provisions in Korea are distributed across multiple documents rather than consolidated within a dedicated contingency planning framework for laboratory animal facilities. Existing guidance documents address selected preparedness elements, such as backup systems and responses to unexpected events during facility inspections, while relevant legislation focuses primarily on disaster prevention associated with hazardous materials or infectious agents [19, 20]. As a result, explicit guidance addressing the continuity of routine animal care and welfare during emergencies remains relatively limited.

Overall, the comparative review demonstrated that emergency preparedness has increasingly been recognized as an institutional responsibility across jurisdictions. However, substantial differences remain in the regulatory mechanisms, scope, and level of specificity through which preparedness requirements are implemented.

#### Comparison of core elements in emergency preparedness programs

Despite these differences in regulatory approach, several common elements emerged across jurisdictions, including advance planning, clearly defined roles and responsibilities, veterinary involvement, communication systems, personnel training, and procedures for maintaining animal care during service disruptions (Table 2). These recurring components formed the basis for the development of the integrated framework proposed in this study.

**Table 2:** Comparison of core elements in emergency programs

Elements	Regulatory Instrument					
	US :USDA	US :PHS	EU	CA :CCAC	KR :MAFRA	KR :KFDA
Risk assessment	O	O	△	O	ㅡ	ㅡ
System loss response	O	O	O	O	O	ㅡ
Resource management	O	O	△	O	ㅡ	ㅡ
Facility security	△	O	O	O	ㅡ	ㅡ
Animal Disposition	O	O	O	O	ㅡ	ㅡ
Infection prevention	ㅡ	ㅡ	O	△	ㅡ	O
Veterinary Care	O	O	O	O	ㅡ	ㅡ
Personnel Training	O	O	O	O	O	ㅡ
Institutional coordination	O	O	O	O	ㅡ	ㅡ
Emergency communication	O	O	O	O	ㅡ	ㅡ
Periodic review and program evaluation	O	O	△	O	ㅡ	ㅡ

O, explicitly addressed in the regulation or guidance document

△, indirectly addressed or partially described

–, not identified

The extent to which these elements were addressed varied among regulatory systems. Core operational elements of emergency preparedness programs were identified through a comparative review of major regulatory and guidance frameworks for laboratory animal facilities, including the Animal Welfare Regulations, Public Health Service Policy, the Guide for the Care and Use of Laboratory Animals, Directive 2010/63/EU, FELASA recommendations, and CCAC guidelines. The United States and Canada presented the most comprehensive coverage, with guidance documents addressing a broad range of operational and organizational considerations, including risk assessment, contingency planning, veterinary oversight, emergency communication, institutional coordination, and periodic program review. In the European framework, recent amendments to Directive 2010/63/EU established contingency planning as a regulatory requirement, while FELASA guidance provided additional recommendations regarding veterinary responsibilities, personnel training, and emergency preparedness at the institutional level. In contrast, Korean regulations and guidance documents addressed selected aspects of emergency preparedness, particularly infrastructure protection, personnel training, and disaster prevention measures, but provided comparatively limited guidance regarding the operational management of animals during emergencies.

Several elements were identified consistently across nearly all jurisdictions. These included system loss response, veterinary involvement, institutional coordination, emergency communication, and personnel training. Together, these elements reflect a common recognition that effective emergency preparedness requires both technical measures to maintain critical services and organizational mechanisms to support timely decision-making and coordinated response. By comparison, risk assessment, resource management, and periodic program evaluation showed greater variation among jurisdictions, reflecting differences in regulatory emphasis, institutional expectations, and implementation strategies.

Notably, veterinary care was among the most consistently represented elements across the international frameworks reviewed. Although the scope of responsibility differed between jurisdictions, veterinary personnel were generally expected to contribute to animal welfare assessment, clinical decision-making, and the development or implementation of emergency response procedures. This recurring role highlights the importance of veterinary oversight as a mechanism for integrating animal welfare considerations into broader emergency preparedness and response activities.

**Fig. 1:**
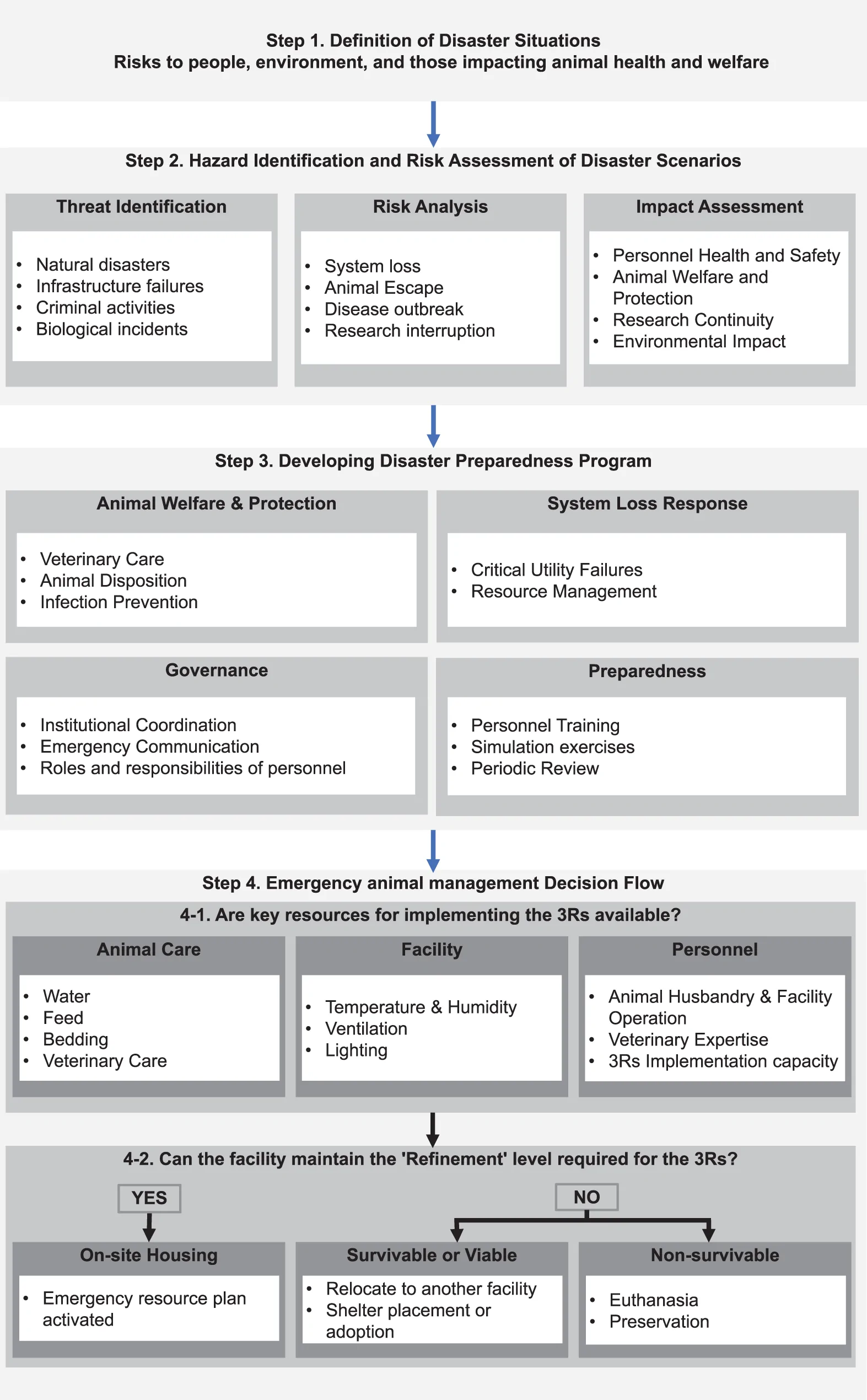
A four-step framework for emergency preparedness and welfare-informed decision making in laboratory animal facilities

Based on the recurring elements identified through the comparison, emergency preparedness programs were organized into three functional domains: facility operational continuity, animal care and welfare, and institutional response capacity. These domains served as the foundation for the Integrated Emergency Preparedness Framework presented in Fig. 1.

**Risk assessment**: Identification and evaluation of potential emergency scenarios and their likely impact on animal welfare, facility operations, personnel safety, and research activities to support preparedness planning and decision-making; **System Loss Response**: Planned actions to maintain or restore animal care and environmental control following failure of critical facility systems (e.g., HVAC, electricity, or water supply); **Resource Management**: Planning and allocation of essential supplies, equipment, and personnel required to sustain animal care and facility operations during emergencies; **Facility Security**: Measures to maintain secure facility operation, control access, prevent unauthorized entry, and maintain operational control of the facility to protect personnel, animals, and infrastructure during emergencies; **Animal Disposition**: Predefined strategies for managing animals during emergencies, including continued care, relocation, humane euthanasia, or biological preservation, based on animal welfare considerations and resource availability; **Veterinary Care**: Provision of clinical care, welfare assessment, and veterinary oversight to support animal welfare consideration and humane decision-making during emergencies; **Personnel Training**: Training programs that prepare personnel to respond effectively and safely during emergencies, including role-specific responsibilities and animal welfare considerations; **Institutional Coordination**: Structured collaboration among researchers, facility leadership, veterinary staff, and external agencies to ensure a coordinated and authorized emergency response; **Emergency Communication**: Protocols for disseminating critical information, establishing communication chains, and ensuring timely updates among staff and external partners during emergencies; **Periodic Review and Program Evaluation**: Regular review, testing, and updating of emergency preparedness programs to ensure continued effectiveness and readiness.

#### Decision framework for integrating animal welfare oriented emergency preparedness

Emergency preparedness programs across the jurisdictions reviewed included a variety of operational, veterinary, and organizational elements; however, the structure and emphasis of these elements varied among regulatory systems. Although common components were identified, they were often distributed across multiple regulations, guidance documents, and institutional procedures rather than presented within a single operational framework.

To integrate these recurring elements into a practical structure for laboratory animal facilities, a four-step framework for emergency preparedness and welfare-informed decision making was developed (Fig. 1). Step 1 expands the definition of emergencies to encompass a broad range of foreseeable events that may affect animal care, facility operations, personnel, and research activities. Step 2 incorporates risk identification and assessment to evaluate potential hazards, vulnerabilities, and their likely consequences for animal welfare and institutional operations. Step 3 organizes preparedness activities into four operational domains: Animal Welfare and Protection, System Loss Response, Governance, and Preparedness. Together, these domains encompass the principal elements identified across the regulatory frameworks reviewed, including veterinary care, animal disposition, resource management, institutional coordination, communication, personnel training, and program evaluation. Effective emergency preparedness requires advance planning and predefined criteria for animal disposition under different emergency scenarios [1]. Step 4 provides a structured decision-making process for animal management during emergencies. The framework first evaluates the availability of essential resources required to maintain animal care, facility operations, and personnel support. It then considers whether an acceptable level of refinement can be maintained under the prevailing conditions. Based on this assessment, animals may remain on site, be relocated to an alternative facility, or undergo humane euthanasia or preservation, depending on welfare considerations, resource availability, and veterinary judgment.

Rather than introducing new regulatory requirements, the framework is intended to provide a practical structure through which institutions can integrate animal welfare considerations into emergency preparedness activities. By doing so, it provides a more systematic approach to managing laboratory animals during emergencies while facilitating the integration of animal welfare considerations and veterinary input into preparedness, response, and operational decision-making.

#### Proposal for a practical checklist for emergency preparedness

Based on the regulatory review and comparative analysis, a practical emergency preparedness checklist for laboratory animal facilities was developed (Table 3). The proposed checklist is organized into six domains: governance and responsibility structure, animal care and veterinary oversight, infrastructure and resource management, training and program validation, communication and coordination, and security and personnel safety. Within these domains, the checklist incorporates key elements, including risk assessment, contingency planning, veterinary involvement, animal disposition, personnel training, emergency communication, institutional coordination, and periodic program review. “Must” items represent foundational elements that were consistently identified across multiple international regulatory frameworks (Table 2) and considered essential for maintaining core emergency preparedness functions of emergency preparedness programs regardless of institutional size or resources. “Should” items represent additional measures recommended by selected guidance documents or current best practices that may further strengthen institutional preparedness according to facility characteristics, available resources, and anticipated emergency scenarios. By distinguishing between core preparedness elements and additional recommended measures, the checklist is intended to provide a practical and flexible framework that can be adapted to different facility characteristics, available resources, and anticipated emergency scenarios.

**Table 3:** Integrated emergency preparedness checklist for laboratory animal facility

Laboratory Animal Facility Emergency Preparedness Checklist (Animal Welfare–Centered Framework)			
Domain	Item	Required (must)	Recommended (should)
1. Governance & Responsibility Structure	Has a facility-specific risk assessment been conducted to identify foreseeable emergency scenarios and their potential impact on animal care, personnel safety, and facility operations?	✔	
	Is the emergency preparedness program formally documented (e.g., SOPs or an emergency response plan)?	✔	
	Is an institution-wide crisis or emergency management framework clearly defined?		✔
	Are roles and responsibilities clearly assigned, including institutional leadership, facility management, and the attending veterinarian?	✔	
	Is the decision-making authority during emergencies explicitly described?	✔	
	Is the role of the IACUC integrated into emergency planning and response?	✔	
	Is the program reviewed and updated on a regular basis (e.g., annual or semiannual review)?	✔	
2. Animal Care & Veterinary Oversight	Does the program ensure continuity of basic animal care (food, water, environmental control) during emergencies?	✔	
	Are veterinary care, treatment prioritization, and euthanasia criteria defined in advance?	✔	
	Are predefined decision criteria available for animal disposition, continued care, or humane euthanasia under different emergency scenarios?		✔
	Are humane endpoints and pain/distress minimization explicitly addressed?	✔	
	Is the role and authority of the attending veterinarian clearly defined during emergency decision-making?	✔	
	Is preservation of critical research resources (e.g., cryopreservation of germplasm or biological materials, where applicable) considered?		✔
3. Infrastructure & Resource Management	Are contingency plans in place for critical system failures (power, water, HVAC)?	✔	
	Are backup systems, alternative housing options, and essential equipment identified and accessible?		✔
	Are essential personnel identified with plans for staffing shortages or absenteeism?	✔	
	Are minimum stock levels defined for critical supplies (feed, bedding, medications)?		✔
	Are agreements or pre-arrangements established for resource sharing or animal relocation, if needed?		✔
4. Training, Simulation & Program Validation	Are emergency preparedness trainings provided to all relevant personnel or designated essential staff?	✔	
	Do training programs incorporate animal welfare and ethical decision-making considerations?	✔	
	Are drills or tabletop exercises conducted periodically to test emergency procedures?		✔
	Are outcomes of training and simulations documented and used to improve the program?		✔
	Is emergency response training included in onboarding or continuing education programs?	✔	
5. Communication, Coordination & Social Trust	Are internal communication pathways and emergency contact lists clearly defined and maintained?	✔	
	Are coordination mechanisms established with external entities (emergency responders, law enforcement, regulatory authorities)?	✔	
	Are principles for external communication during emergencies defined in advance?		✔
	Is a designated spokesperson or communication lead identified?		✔
	Are core messages prepared to explain animal welfare considerations and ethical decision-making during emergency situations?		✔
6. Security and Personnel Safety	Are procedures in place to prevent and respond to animal escape during emergency situations?	✔	
	Are access control measures and facility lockdown procedures established to maintain security during emergencies?	✔	
	Are response plans established for incidents involving unauthorized entry, vandalism, or violence affecting the animal facility?		✔
	Are measures in place to ensure the protection and safety of personnel during emergency situations?	✔	

This approach recognizes the diversity of laboratory animal facilities while maintaining a consistent framework for preparedness planning. Rather than prescribing a single response model, the checklist is intended to serve as a practical tool for developing facility-specific standard operating procedures (SOPs), identifying potential gaps in preparedness, and supporting consistent decision-making during emergency situations.

## Discussion

This study identified substantial variation in how emergency preparedness is defined and implemented across regulatory frameworks governing laboratory animal facilities. Despite differences in regulatory structure and enforceability, several common elements were consistently observed, including contingency planning, veterinary involvement, communication systems, personnel training, and procedures for maintaining animal care during service disruptions. These observations indicate broad international agreement regarding the core components of emergency preparedness programs. However, the reviewed frameworks differed in how these preparedness elements were translated into operational decision-making during emergencies. Decisions involving continued animal care, relocation, resource preservation, or humane euthanasia varied in their level of specificity across regulatory systems, and practical approaches for institutional implementation were not consistently articulated. This indicates a need for approaches that help institutions connect preparedness planning with animal-related decision-making under crisis conditions.

Emergency preparedness programs have historically focused on maintaining human safety, operational continuity, and the integrity of critical infrastructure [21–23]. Across the regulatory frameworks reviewed, preparedness measures were commonly directed toward sustaining essential services such as power, ventilation, water supply, communication systems, and staffing capacity. However, because laboratory animals are highly dependent on controlled environments and continuous human care, disruptions in facility operations can rapidly affect animal health and welfare. Historical disasters illustrate both this vulnerability and the value of advanced preparation. Tropical Storm Allison (2001) and the Great East Japan Earthquake (2011) resulted in the loss or humane euthanasia of tens of thousands of laboratory animals despite the existence of emergency plans [9, 13]. In contrast, Fukushima Medical University was able to minimize animal losses and limit the need for euthanasia through preparedness measures such as anti-tipping facility design and advance planning for hypothermia management [24]. These cases indicate that emergency outcomes are influenced not only by the magnitude of a disaster but also by the preparedness measures established before the event.

Accordingly, emergency situations often require decisions that extend beyond facility recovery alone and include continued animal care, relocation, preservation of valuable animal resources, or humane euthanasia. Under conditions of uncertainty and resource limitation, such decisions may require balancing welfare priorities with scientific and institutional considerations. This reinforces the importance of incorporating welfare considerations and veterinary input into preparedness planning before emergencies occur [4, 25].

Traditionally, the 3Rs principles (Replacement, Reduction, and Refinement) have been applied primarily during protocol design and routine animal use. However, emergency situations also require ethical decisions regarding continued animal care, animal relocation, preservation of valuable animal resources, and humane euthanasia. In this context, the 3Rs may provide an overarching set of ethical principles to support welfare-oriented decision-making throughout emergency preparedness rather than being confined to routine animal research. For example, Reduction may support advance planning strategies aimed at minimizing avoidable animal losses through colony management and the preservation of valuable animal resources. Refinement may guide the development of predefined decision criteria for continued animal care, veterinary intervention, animal relocation, and humane euthanasia to minimize pain and distress when standard husbandry conditions can no longer be maintained.

Importantly, the role of the 3Rs begins before an emergency occurs. During the preparedness phase, these principles may inform risk assessment, advance planning, and the establishment of predefined decision criteria for continued animal care, relocation, preservation of valuable animal resources, and humane euthanasia. Establishing these criteria in advance may improve consistency and reduce ethical uncertainty when rapid decisions become necessary. During emergency response, these predefined strategies may provide a structured basis for evaluating available options while taking animal welfare, veterinary judgment, and operational constraints into account. From this perspective, emergency preparedness should not be viewed solely as a means of maintaining institutional operations. Rather, it provides a practical framework that facilitates the incorporation of established animal welfare principles, including the 3Rs, into emergency planning and operational decision-making. To facilitate implementation, this study developed a checklist (Table 3 and Supplementary Table S1) based on a two-tiered classification system consisting of “Required” and “Recommended” items. This structure was designed to allow adoption across institutions operating under different regulatory environments, resource constraints, and facility characteristics.

In addition to commonly recognized preparedness elements, the checklist incorporates broader organizational considerations, including governance structures, facility security, communication pathways, and institutional coordination. These factors may be particularly relevant during emergencies that require difficult decisions regarding animal management, where clearly defined responsibilities and effective communication can support timely and accountable decision-making [1, 26]. The checklist may therefore serve as a practical reference for developing and reviewing institutional standard operating procedures (SOPs), personnel training programs, and preparedness assessments.

This study has several limitations. First, because the analysis was based primarily on regulatory and guidance documents, it may not fully capture how emergency preparedness programs are implemented in practice at the institutional level. Second, the comparative review was limited to four jurisdictions and therefore may not reflect the full range of international approaches to emergency preparedness in laboratory animal facilities. Third, as regulatory documents are periodically revised, the comparative findings represent the frameworks as they stood at the time of analysis. Future research would benefit from empirical case studies, institutional surveys, and evaluations of preparedness programs to further assess the applicability of the proposed framework. Additional research may also help clarify how welfare considerations and the 3Rs can be incorporated across different emergency scenarios, facility types, and species-specific contexts.

## Conclusions

This study demonstrates that, although emergency preparedness is increasingly recognized as an institutional responsibility across jurisdictions, substantial differences remain in how preparedness requirements are translated into practical decision-making for laboratory animal care. To address the gap between preparedness planning and operational decision-making, we propose an integrated emergency preparedness framework that links risk assessment, preparedness planning, resource evaluation, and operational decision-making within a single process. The framework is not intended to introduce new regulatory requirements, but rather to connect widely recognized preparedness elements within a coherent operational structure that may support more consistent decision-making at the institutional level.

The checklist developed in this study may serve as a practical reference for developing and reviewing institutional standard operating procedures (SOPs), personnel training programs, and preparedness assessments. Although emergency preparedness cannot ensure animal welfare under every circumstance, it can promote more consistent, transparent, and welfare-oriented decisions during crises [12, 27].

## Electronic supplementary material

Below is the link to the electronic supplementary material.

Supplementary Material 1

## Data Availability

No datasets were generated or analysed during the current study.

## Abbreviations

AWR: Animal Welfare Regulations
EU: European Union
FELASA: Federation of European Laboratory Animal Science Associations
HVAC: Heating, Ventilation, and Air Conditioning
IACUC: Institutional Animal Care and Use Committee
PHS: Public Health Service
SOPs: Standard Operating Procedures
USDA: United States Department of Agriculture.
