# Supplementary material for: Reframing emergency preparedness from facility survival to laboratory animal welfare
Source: Lab Anim Res. 2026 Sep 8;42:35. doi: 10.1186/s42826-026-00295-5 (PMC13551745; doi:10.1186/s42826-026-00295-5)
Supplement: Supplementary file 1 [file 42826_2026_295_MOESM1_ESM.docx]

**Supplementary Table 1. Checklist for Effective Emergency Preparedness Programs in Laboratory Animal Facilities**

| Domain | Checklist Item | Required (must) | Recommended (should) | Source |
| --- | --- | --- | --- | --- |
| Emergency Definition & Risk Assessment | Emergency situations affecting animal care and facility operations are formally defined | ✔ |  | Guide; CCAC; PHS |
|  | Facility-specific hazards (natural disasters, system failure, disease outbreak, security breach) are identified | ✔ |  | Guide; CCAC |
|  | Risk assessments consider impacts on human safety, animal welfare, and research resources | ✔ |  | Guide; AAALAC |
|  | Risk scenarios relevant to local environmental and institutional conditions are documented |  | ✔ | CCAC; FELASA |
| System Loss Response | Response procedures for loss of critical systems (electricity, HVAC, water supply) are established | ✔ |  | Guide; CCAC |
|  | Backup systems or contingency procedures are available for maintaining animal care | ✔ |  | Guide; CCAC |
| Resource Management | Essential supplies (feed, bedding, PPE, medical supplies) are secured for emergency situations | ✔ |  | Guide; CCAC |
|  | Contingency plans exist for disruption of supply chains |  | ✔ | FELASA; CCAC |
| Animal Disposition | Procedures for animal relocation, continued care, or humane euthanasia during emergencies are defined | ✔ |  | Guide; CCAC; AVMA |
|  | Alternative housing or transfer arrangements with other facilities are considered |  | ✔ | CCAC |
| Veterinary Care | Veterinary oversight and medical care for animals during emergencies are ensured | ✔ |  | Guide; CCAC |
|  | Veterinary staff participate in emergency planning and decision-making |  | ✔ | Guide; CCAC |
| Personnel Training | Staff roles and responsibilities during emergencies are clearly defined | ✔ |  | Guide; CCAC |
